# The Influence of Model Orientation on the Surface Roughness of Polymeric Models Produced by FFF, mSLA, PJ, and SLS Methods

**DOI:** 10.3390/ma18245600

**Published:** 2025-12-12

**Authors:** Anna Bazan, Paweł Turek, Grzegorz Budzik, Piotr Niesłony, Roman Grygoruk, Przemysław Siemiński

**Affiliations:** 1 Faculty of Mechanical Engineering and Aeronautics, Rzeszów University of Technology, 35-959 Rzeszów, Poland; pturek@prz.edu.pl (P.T.); gbudzik@prz.edu.pl (G.B.); 2 Faculty of Mechanical Engineering, Opole University of Technology, Mikołajczyka 5 Str., 45-001 Opole, Poland; p.nieslony@po.edu.pl; 3 Faculty of Mechanical and Industrial Engineering, Warsaw University of Technology, Narbutta 85 Str., 02-524 Warsaw, Poland; roman.grygoruk@pw.edu.pl; 4 Faculty of Automotive and Construction Machinery Engineering, Warsaw University of Technology, Narbutta 84 Str., 02-524 Warsaw, Poland; przemyslaw.sieminski@pw.edu.pl

**Keywords:** additive manufacturing, orientation, surface roughness, optical measurements, polymer, vat polymerization, powder bed fusion, material extrusion, material jetting

## Abstract

The research methodology involved creating a 3D sample model that featured both flat and cylindrical surfaces inclined at angles ranging from 0° to 90° relative to the XY plane. The study investigated the surface topography of additively manufactured samples produced using various technologies, including Fused Filament Fabrication (FFF), masked Stereolithography (mSLA), PolyJet (PJ), and Selective Laser Sintering (SLS). The focus was on how material type, print angle, and measurement location influenced the results. The materials used in the study included PLA, PETG, acrylic resins, PA2200, and VeroClear. Due to the optical properties of the materials used, measurements were carried out on replicas that were prepared using a RepliSet F5 silicone compound from Struers. Consequently, a methodology was developed for measuring surface roughness using the Alicona microscope based on these replicas. A 10× objective lens was used during the measurements, and the pixel size was 0.88 µm × 0.88 µm. Each time, an area of approximately 1 mm × 4 mm was measured. The lowest roughness values were observed for mSLA samples (Sa = 6.72–8.54 µm, Spk + Sk + Svk = 33.36–42.16 µm), whereas SLS exhibited the highest roughness (Sa = 27.86 µm, Spk + Sk + Svk = 183.79 µm). PJ samples exhibited intermediate roughness with significant anisotropy (Sa = 11.65 µm, Spk + Sk + Svk = 72.1 µm), which was strongly influenced by the print angle. FFF surfaces showed directional patterns and layer-dependent roughness, with the Sa parameter being the same (12.44 µm) for both PETG and PLA materials. The steepest slopes were observed for SLS surfaces (Sdq = 7.67), while mSLA exhibited the flattest microstructure (Sdq = 0.48–0.89). Statistical analysis confirmed that material type significantly influenced topography in mSLA, while print angle strongly affected PJ and FFF (although for FFF, further studies would be beneficial). The results of the research conducted can be used to develop a methodology for optimizing the printing process to achieve the required geometric surface structure.

## 1. Introduction

Additive manufacturing (AM) techniques are rapidly emerging as advanced technologies for creating complex models [1,2]. During the AM process, material is deposited layer by layer until the final model is fully realized [3]. Today, there is a wide range of AM methods available on the market. In collaboration with the ISO (International Organization for Standardization) and ASTM (American Society for Testing and Materials), two key standards have been established: ISO/ASTM 52900 [4] and ISO/ASTM 52910 [5]. These standards outline the additive techniques currently in practice. To date, seven distinct processes have been officially defined for shaping objects through AM. In AM technologies, metal alloys are still used, but the use of polymer materials is growing because of improvements in their properties. Based on data from the Wohlers Report 2025 and related market analyses, the polymer materials segment is key to the additive manufacturing industry [6]. It is estimated that the revenue from the sale of polymer materials (powders, resins, filaments) reached approximately $1.5 billion USD in 2024 [6]. If we accept these estimates, the share of polymer materials in the total materials market ($4.4 billion USD) was approximately 34% in 2024. This rapid growth is driven by the increasing demand for high-performance thermoplastics and the development of continuous fiber polymer composites, signaling a shift from prototyping to the production of final parts [6].

Additive technologies are frequently used to create functional models using polymer materials, which necessitates strict quality standards for the surface texture. As of now, no single additive technique has emerged as the leader in industrial applications [7]. This situation is influenced by various factors, one of which is the quality of the surface texture, particularly concerning surface roughness [8,9]. Surface roughness significantly influences the physical and functional properties of parts. From a mechanical perspective, it can affect friction and wear [10], adhesive characteristics [11], and material fatigue [8]. Additionally, it impacts both esthetic and functional aspects of the components [12]. Therefore, minimizing surface roughness is a primary goal in optimizing the additive manufacturing process. The surface roughness of a product can vary depending on the additive manufacturing technique used. For instance, products made using Vat Polymerization (VPP) techniques typically exhibit high-quality surfaces [13,14]. In contrast, surfaces created through Powder Bed Fusion (PBF) technology tend to be more matte and porous [15,16]. Additionally, with Material Extrusion (MEX) technology, surface roughness can vary significantly based on the orientation of the model within the 3D printer [17,18]. Several factors contribute to this variability, with the most significant being the type of additive manufacturing source (e.g., nozzle, laser, projector, or ultraviolet lamp) [19,20], the material used [21], and the 3D printing parameters related to layer thickness [22]. However, one parameter consistently impacts surface roughness across all manufacturing technologies: the orientation of the model in the 3D printer [23,24]. The degree of this impact varies with different additive manufacturing technologies and remains broadly understood.

Previous research on the surface roughness of polymer models produced using additive manufacturing methods has highlighted key factors that impact the quality and accuracy of 3D printing. While research is currently being conducted to assess the effects of orientation changes, most of this work focuses on MEX technology [25,26]. Studies have shown that reducing layer thickness significantly decreases the staircase effect, resulting in smoother surfaces. Additionally, it has been confirmed that the orientation of the model plays a crucial role in surface roughness, particularly on curved and inclined surfaces, as it affects the distribution of “steps.” Moreover, comparisons are being made between different measurement methods [27,28]. Findings indicate that optical methods are generally more effective for evaluating the topography of 3D printed surfaces compared to contact methods, especially for determining the characteristics of irregular surfaces [29,30]. A comparison of roughness across various technologies reveals that VPP and Material Jetting (MJ) typically produce much smoother surfaces than MEX, attributable to their smaller layer and droplet sizes [31]. In PBF technologies, surface roughness is more closely related to the size of the powder grains rather than the layering effect [32]. Additionally, attention is being given to mathematical and statistical models that can predict surface roughness based on printing parameters [33,34]. Efforts are ongoing to develop more advanced machine learning algorithms that automatically optimize the 3D printing process.

Despite advancements in research on assessing surface roughness of polymer material models, there remain several knowledge gaps, particularly regarding the evaluation of surface roughness related to:

- Limited comparative analysis across multiple AM technologies: Most existing studies focus on a single additive manufacturing technology or material, making it difficult to directly compare surface roughness behavior across different processes. Moreover, measured surface parameters are highly dependent on the measurement method and the size of the analyzed area, which further complicates cross-study comparisons. There is a lack of systematic investigations that evaluate the combined effects of material type, layer thickness, and print orientation on surface topography parameters using a consistent measurement methodology across several AM technologies.
- Insufficient understanding of local surface variability–While average roughness values are often reported, there is limited knowledge about how surface roughness parameters vary within a single printed part depending on local geometry (e.g., flat vs. curved regions) and measurement location. This gap hampers accurate prediction of functional surface performance.
- Scarcity of quantitative models linking orientation, material, and topography: Existing models often fail to capture the complex interactions between print angle, layer stacking, and material-specific properties that influence both height and slope of surface irregularities. Consequently, guidelines for selecting optimal print orientation to minimize roughness remain incomplete.

This deficiency significantly hinders the commercialization of finished products produced through AM, particularly in industries such as aviation and automotive.

This study provides a systematic and unified comparison of surface topography across four polymer AM technologies (FFF, mSLA, PJ, SLS) using a single multi-geometry test artifact and high-resolution non-contact measurements on silicone replicas. By analyzing the effects of printing angle, surface curvature, and measurement location within one consistent metrological framework, the work offers new comparative insight into how process characteristics shape surface height and slope. These findings address important gaps in cross-technology roughness evaluation and support more informed selection of AM processes for functional applications.

## 2. Materials and Methods

**Figure 1:**
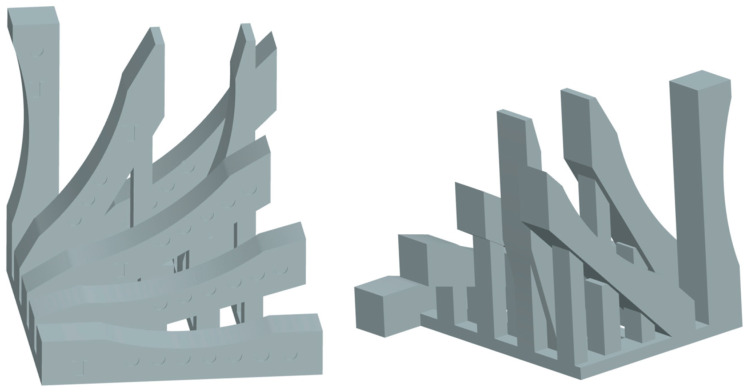
The developed research 3D-CAD model.

The research sample was developed and modeled using Catia V5 software. The sample, with overall dimensions of 18 mm × 4 mm × 5 mm, features both flat and cylindrical surfaces. During the modeling process, the sample was inclined to the x–y plane at angles of 0°, 15°, 30°, 45°, 60°, 75°, and 90°. A total of seven models, each representing one inclination angle, were combined into a single CAD assembly (Figure 1).

When exporting the data to the Stereolithography (STL) file, the Tessellation function from the STL Rapid Prototyping module was used. This function allows control over the quality of the faceted surface generation based on two parameters: sag and step. The sag parameter defines the acceptable deviation of the generated wall of the 3D STL model from the geometry of the Computer-Aided Design (CAD) file. Meanwhile, the step parameter directly influences the length of the triangle sides. To generate the 3D STL model, it was decided to set the values for both the sag and step parameters to 0.005 mm. This choice strikes a balance between the accuracy of the 3D STL model generation and the capabilities of currently available 3D printers. Consequently, errors that could occur during the data export from CAD to STL format will be minimized during the additive manufacturing process. Four additive technologies were used in the model manufacturing process (Table 1).

**Figure 2:**
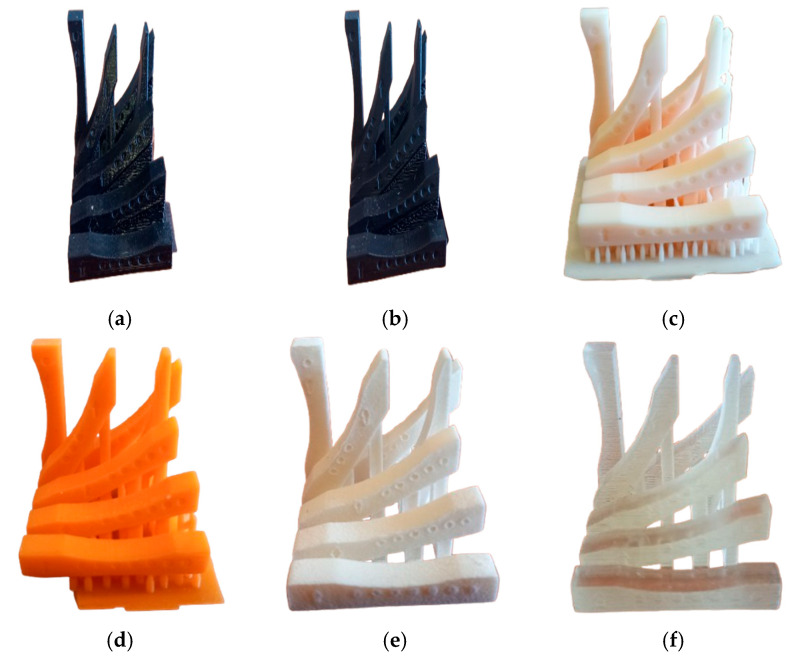
Additively manufactured models: (**a**) PETG; (**b**) PLA; (**c**) Phrozen Aqua-Ivory 4K; (**d**) Phrozen Neon Pumpkin; (**e**) PA2200; (**f**) VeroClear.

Based on the 3D-STL model developed, using 3D printers, physical models were produced using PETG (Figure 2a), PLA (Figure 2b), Phrozen Aqua-Ivory 4K (Figure 2c), Phrozen Neon Pumpkin (Figure 2d), PA2200 (Figure 2e), and VeroClear (Figure 2f) materials.

Measurements of the surface topography of the printed models were carried out using an Alicona InfiniteFocus G4 microscope (Alicona Imaging GmbH, Raaba-Grambach, Austria). Due to the optical properties of the materials from which the samples were made, the measurements were carried out on replicas. The decision to use replicas was made after conducting direct measurements on the test specimens. For nearly all materials (except PETG and Phrozen Neon Pumpkin), the proportion of non-measurable points exceeded 50%, which is far beyond the acceptable threshold for reliable topography acquisition. Such a high percentage of missing data leads to fragmented or distorted surface reconstruction, and therefore the direct measurements were classified as insufficient in quality. Problems with carrying out direct measurements could have resulted from:

- Transparent and translucent Materials (like VeroClear) exhibit high light transmission (>50%). This causes subsurface scattering, meaning the measurement light penetrates the material instead of reflecting precisely from the surface. This effect prevents the Alicona system from accurately determining the focal plane (maximizing contrast) and results in false height readings from a ‘virtual’ surface beneath the actual topography.
- The powder-based PA2200 (SLS), composed of sintered particles, acts as a strong diffuser (Lambertian-like reflector). This creates numerous shadows and chaotic micro-reflections, causing the Alicona system to receive an insufficient or highly scattered signal along the optical axis, leading to a high percentage of non-measurable points (‘dark spots’) and fragmented data acquisition.
- Low optical contrast: light-colored materials such as white PA2200, beige PLA, or light-pink Phrozen Aqua-Ivory exhibit very high diffuse reflectance (albedo). The surface reflects light almost uniformly in all directions, resulting in small intensity differences between reflections from peaks and valleys. This low signal variability reduces the local contrast required for the proper operation of the Focus Variation algorithm.

**Figure 3:**
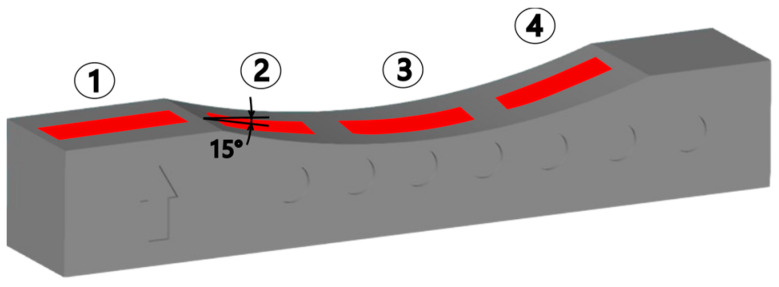
Location of measurement areas.

To prepare the replicas, RepliSet F5 silicone compound from Struers (Ballerup, Denmark), dedicated for surface roughness measurements, was used. As stated by the manufacturer, the material used was a black silicone rubber that can reproduce features finer than 0.1 µm. The quality of the surface reproduction achieved with this replication system is presented in [35]. For each model, measurements were taken in four locations (Figure 3). The angle between surface in location 1 and the tangent to surfaces 2 and 4, determined at the center of the measurement area, was 15°. For each sample type, a total of 28 surfaces were measured—corresponding to seven inclination angles (0–90°) and four measurement locations per angle. A 10× objective lens was used during the measurements, and the pixel size was 0.88 µm × 0.88 µm. Each time, an area of approximately 1 mm × 4 mm was measured. The measurements were performed using white light. The measurement data were processed in the SPIP 6.4.2 software, and surface topography parameters were determined. Statistical analyses were performed using Python 3.13 programming libraries. In all tests, the significance level was set at 0.05. To account for multiple comparisons, Bonferroni correction was applied within each set of analyses for a given sample type or surface texture parameter. This correction is commonly used in the literature when the number of comparisons is relatively small, as it provides a simple and conservative way to control the family-wise error rate and reduce the risk of false positives. The significance level, corrected using the Bonferroni method, was denoted as αB.

## 3. Results

### 3.1. Influence of Samples’ Type on Surface Topography

#### 3.1.1. Visual Assessment of Topography Maps

**Figure 4:**
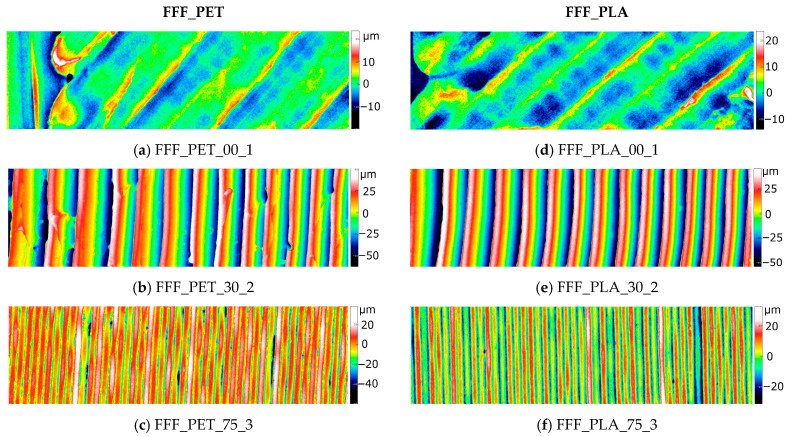
Representative 2D topography maps of FFF samples at different inclination angles and measurement locations: (**a**–**c**) PETG samples (0°/loc.1; 30°/loc.2; 75°/loc.3) and (**d**–**f**) PLA samples (0°/loc.1; 30°/loc.2; 75°/loc.3). Measurement area: 4 mm × 1 mm.

**Figure 5:**
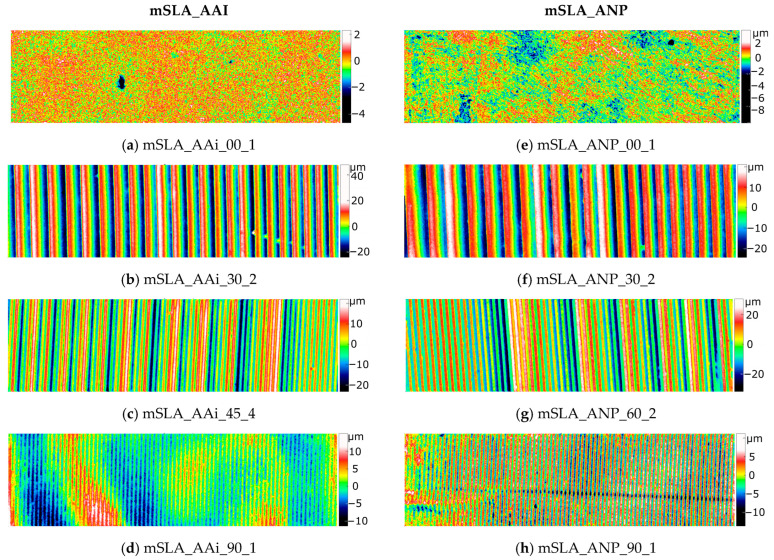
Representative 2D topography maps of mSLA samples at different inclination angles and measurement locations: (**a**–**d**) mSLA_AAI samples (0°/loc.1; 30°/loc.2; 45°/loc.4; 90°/loc.1) and (**e**–**h**) mSLA_ANP samples (0°/loc.1; 30°/loc.2; 60°/loc.2; 90°/loc.1). Measurement area: 4 mm × 1 mm.

**Figure 6:**
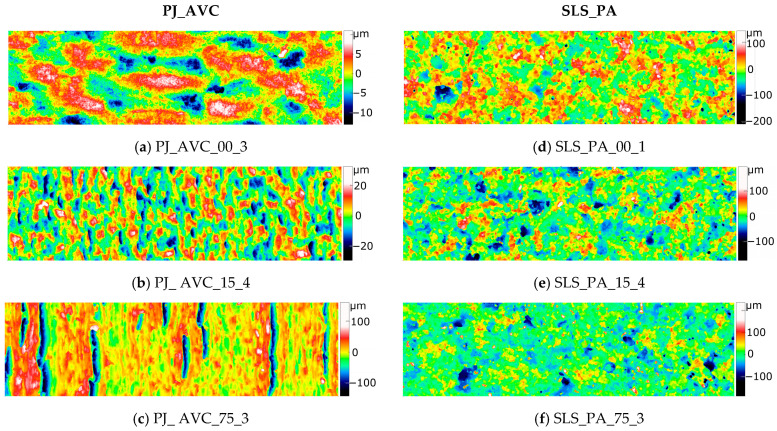
Representative 2D topography maps of PJ_AVC and SLS_PA samples at different inclination angles and measurement locations: (**a**–**c**) PJ_AVC samples (0°/loc.3; 15°/loc.4; 75°/loc.3) and (**d**–**f**) SLS_PA samples (0°/loc.1; 15°/loc.4; 75°/loc.3). Measurement area: 4 mm × 1 mm.

For each type of sample, 28 surface fragments with dimensions of approximately 4 mm × 1 mm were measured. To illustrate the variety of surface structures resulting from the printing angle of a given sample and the nominal surface shape, representative areas were selected from among the 28 maps corresponding to each sample type. The 2D maps of the selected fragments, depicting the variability of topography within each sample type, are presented in Figure 4, Figure 5 and Figure 6.

For all types of samples, except for those produced using the SLS method, it was observed that the topography of the flat surface corresponding to the final printed layer (samples labeled 00_1, images (a) in Figure 4, Figure 5 and Figure 6) differs distinctly from the remaining surfaces, which are composed of a greater number of layers.

The surfaces produced using FFF and mSLA technologies (Figure 4) exhibit a directional character. In the case of FFF samples, with relatively large layer thicknesses of 0.08 mm, individual material layers are clearly visible. The peaks corresponding to the filament strands are inclined relative to the mean plane at an angle determined by the slicing of the model into layers.

The topographies of mSLA samples produced from different materials (AAI and ANP) are very similar. In most of the measured surface areas, the individual material layers could be distinguished on the topography maps (Figure 5c,d,f,g). A notable exception were the surfaces printed at a 15° angle relative to the printer’s XY plane. In these cases, a single peak typically consists of three layers, the boundaries between which are not visible on the maps (Figure 5b,f).

Compared to the mSLA and FFF samples, the PJ_AVC samples exhibit a more isotropic character. A measure of isotropy is the Surface Texture Direction Index (Stdi), which ranges from 0 to 1. Surfaces with a clearly dominant direction have Stdi values close to 0, while isotropic surfaces have Stdi values close to 1. The PJ_AVC samples show a higher Stdi value of 0.245 ± 0.043 compared to FFF_PET (0.079 ± 0.016), FFF_PLA (0.085 ± 0.019), mSLA_AAI (0.0790 ± 0.0302), and mSLA_ANP (0.090 ± 0.034).

For models printed at small inclination angles, the layered structure is either not noticeable or only slightly visible (Figure 6b). As the surface inclination relative to the printer’s XY plane increases, the layers become more apparent, the structure acquires a directional character, and characteristic—though relatively few—deep, narrow valleys appear (Figure 6c).

All measured surfaces of the SLS_PA samples exhibited an isotropic character, as also indicated by their highest Stdi value of 0.471 ± 0.012. The additive nature of the manufacturing process was not reflected in the surface topography. No significant differences were observed between the analyzed topography maps of these samples. The low peaks representing minor surface irregularities correspond to individual powder grains, while in the 2D images, clusters of grains forming larger, irregularly shaped peaks are more clearly visible. The dominant valleys also exhibit dimensions substantially exceeding the size of a single grain.

#### 3.1.2. Analysis of Topography Parameters

**Figure 7:**
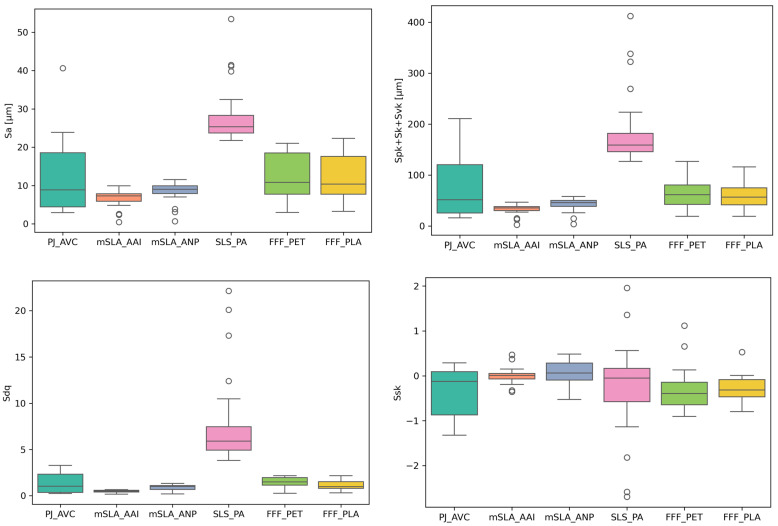
Boxplots showing the dependence of topography parameters on sample type.

Table 2 summarizes the mean values, standard deviations (std), and coefficients of variation (CV) calculated from 28 measurements for each type of sample. A graphical representation of the data is provided in Figure 7.

The Sa parameter and the sum Spk + Sk + Svk indicate surface roughness height—Sa represents the average roughness height, while the sum Spk + Sk + Svk reflects the extreme height differences on the surface [36]. The lowest mean values of these parameters were observed for samples produced using the mSLA technology (for both tested materials): Sa = 6.72 µm (mSLA_AAI) and 8.54 µm (mSLA_ANP), while Spk + Sk + Svk = 33.36 µm and 42.16 µm, respectively.

Despite having a smaller layer thickness, PJ samples exhibited an Sa value over 70% higher than mSLA_AAI (11.65 µm) and approximately 36% higher than mSLA_ANP. Differences in Spk + Sk + Svk were even more pronounced—the PJ sample reached 72.1 µm, more than double the value of mSLA_ANP and over 115% higher than mSLA_AAI. This is likely due to the deep valleys characteristic of PJ samples. Their presence is more strongly reflected in the parameter associated with the total height range of roughness than in the averaged Sa, as they occupy a relatively small surface area.

The highest values of the Sa (27.86 µm) and Spk + Sk + Svk (183.79 µm) parameters were observed for the SLS powder fusion technology. Although larger layer thicknesses were used in the FFF process, the corresponding values for FFF_PLA (Sa = 12.44 µm, Spk + Sk + Svk = 62.87 µm) and FFF_PET (Sa = 12.44 µm, Spk + Sk + Svk = 58.08 µm) were approximately were 55% lower (Sa) and 66–68% lower (Spk + Sk + Svk) than those of SLS. The greater height differences in SLS surfaces are due not only to the presence of deep pores but also to isolated “aggregates” of fused grains, which form unusually high peaks.

The Ssk parameter, describing the skewness of the height distribution, is close to zero for mSLA samples (0.005–0.07), indicating an approximately normal distribution. For the other technologies, Ssk takes negative values (from −0.26 to −0.83), indicating that most height values are concentrated above the mean, while lower values (associated with valleys) are more dispersed. As the depth and number (or proportion) of depressions in the topography increase, the Ssk value generally decreases, reflecting a height distribution increasingly skewed toward negative values, which corresponds to the predominance of valleys over peaks. Two measured SLS sample surfaces exhibited skewness above 1, which was associated with the presence of the unusual high peaks mentioned earlier.

Analyzing the mean surface slope, Sdq, it can be observed that the steepest roughness inclines are associated with the SLS technology (Sdq = 7.67), which is consistent with the visual assessment of the topography maps. A high Sdq value indirectly indicates a highly developed surface microstructure. The mean Sdq values for FFF and PJ samples (1.17–1.5 and 1.31, respectively) are approximately 4–6 times lower than for SLS. In contrast, mSLA samples exhibit the lowest slope steepness and surface development (0.48–0.89).

Additively manufactured components may exhibit different surface topographies in different regions. In the present study, surface measurements were performed on areas with different nominal shapes (flat and cylindrical) that were printed at various angles. The shape and orientation determine the relative displacement of consecutive layers, which in turn affects the resulting topography and its parameters. Therefore, analyzing the variability of the results can provide insight into how much the surface topography can differ across various regions of an additively manufactured component. The variability analysis did not include the Ssk parameter, as it exhibited relatively small values (near 0) for all samples.

**Figure 8:**
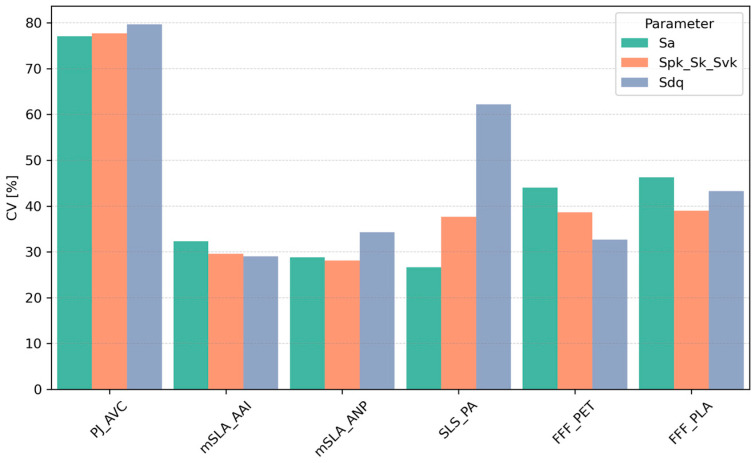
Coefficient of variation (CV) of topography parameters as a function of sample type.

The greatest variability in the Sa, Spk + Sk + Svk, and Sdq parameters, expressed as the coefficient of variation (CV), was observed for PJ samples (CV ≈ 77–80%) (Figure 8). Moderate variability was associated with FFF samples (CV 33–46%, depending on parameter). Samples refered as mSLA exhibited the lowest variability (CV 29–35%). For SLS technology, the CV for Sa and Spk + Sk + Svk was comparable to mSLA and FFF. However, SLS samples showed greater variability in terms of surface slope steepness (Sdq). In Section 3.4 of the article, the analysis of the effects of location and angle on parameter values for each sample type is presented, indicating which of these factors influenced the observed coefficient of variation (CV).

During the variability analysis, it was also examined whether any of the studied topography parameters—Sa, Sdq, and Spk + Sk + Svk—were particularly sensitive to changes within a given sample. To this end, in addition to the coefficient of variation (CV), standardized mean values (z-scores) were analyzed for each sample. The analysis was performed using the Friedman test for paired data (a nonparametric equivalent of ANOVA), with each sample type serving as the pairing factor. The Friedman test applied to both CV and z-score data showed no significant differences between the parameters (αB = 0.008; *p*-values of 1 and 0.85, respectively). This indicates that all three analyzed topography parameters respond to changes within a sample to a similar extent.

The large variability observed in the parameters for the PJ_AVC samples prompted an investigation into the extent to which this variability might depend on the size of the measurement area. For this purpose, the surface texture parameters were also calculated from areas of 2 mm × 1 mm and 1 mm × 1 mm. The coefficient of variation (CV) for the parameters Sa, Spk + Sk + Svk, and Sdq remained at a similar level for different measurement areas, corresponding to areas of 1, 2, and 4 mm^2^: for Sa it was 80.5, 78.1, and 77.1%, for Spk + Sk + Svk 79.6, 78.1, and 77.8%, and for Sdq 79.7, 79.4, and 79.7%, respectively. This indicates a very weak dependence of CV on the measurement area size for the PJ samples. The variability of Sdq was practically the same regardless of the measurement area. The greatest differences were observed for Sa, for which the CV decreased by approximately 3.4% as the measurement area decreased. Interestingly, one might have expected that the variability of Spk + Sk + Svk would depend more strongly on the measurement area than Sa, given that Sa has a more averaging character. It is therefore possible that the observed changes in CV for Sa and Spk + Sk + Svk are small enough that random factors may have had a significant influence on the results.

### 3.2. Influence of Material in mSLA Technology

**Figure 9:**
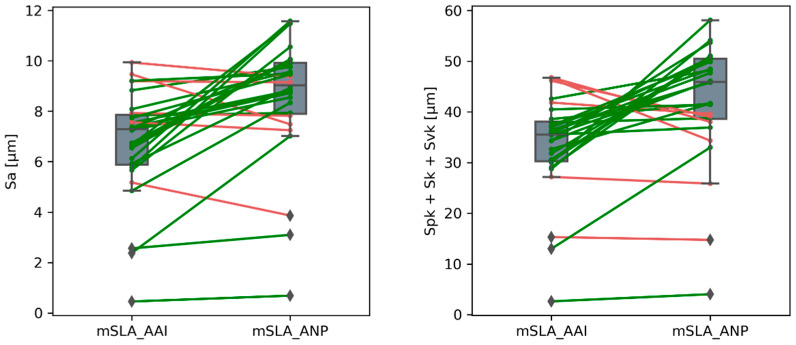

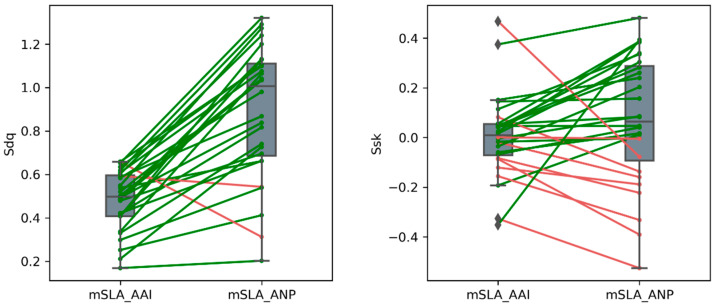
Influence of material in the mSLA method on topography parameter values. Lines connect the same samples (angle + location) in both groups; green indicates an increase, and red indicates a decrease in values for the mSLA_ANP group compared to mSLA_AAI.

Two types of acrylic resin were used to print the mSLA samples. Preliminary analysis of the results (Table 2) indicated that mSLA_ANP samples exhibited higher topography parameter values than mSLA_AAI samples. Statistical tests for paired data (*t*-test or Wilcoxon test, depending on the equality of variances between groups, αB = 0.0125)—with sample angle and measurement location as pairing factors—were conducted to determine whether these differences were statistically significant. A graphical representation of the paired data is shown in Figure 9.

Analysis of the paired differences between mSLA_AAI and mSLA_ANP samples revealed that the mean values of Sa, Spk + Sk + Svk, and Sdq were significantly higher in mSLA_ANP samples than in mSLA_AAI samples (*p*-values for all three tests were <0.0001). For Sa, the mean difference between mSLA_ANP and mSLA_AAI was approximately 1.8 µm; for Spk + Sk + Svk, the mean difference was about 8.8 µm; and for Sdq, 0.42. The only parameter for which the differences were not statistically significant was Ssk (skewness of the height distribution). The mean difference was approximately 0.065, and the Wilcoxon test did not indicate significance (*p* ≈ 0.10), suggesting that the distribution of peaks and valleys was similar for both sample types.

In summary, the changes observed in mSLA_ANP samples relative to mSLA_AAI are clearly reflected in parameters describing roughness height and slope (Sa, Spk + Sk + Svk, Sdq), while they do not significantly affect the asymmetry of the height distribution (Ssk).

### 3.3. Differences Between FFF_PET and FFF_PLA Samples

**Figure 10:**
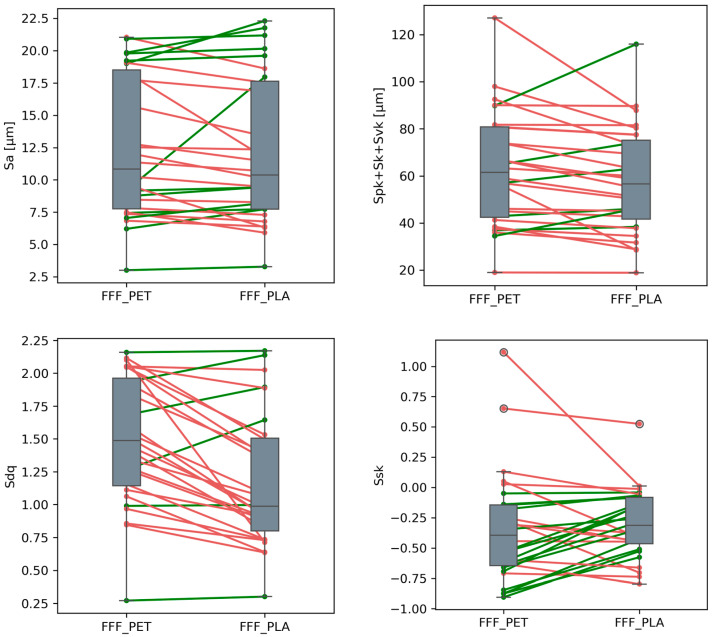
Influence of material and layer thickness in the FFF method on topography parameter values. Lines connect the same samples (angle + location) in both groups; green indicates an increase, and red indicates a decrease in values for the FFF_PLA group compared to FFF_PET.

Two types of filament—PET and PLA—were used to print the FFF samples. Preliminary analysis of the results (Table 2) indicated that FFF_PLA and FFF_PET samples exhibited similar topography parameter values (Sa, Spk + Sk + Svk, and Ssk), and may differ in Sdq values. Statistical tests for paired data (*t*-test or Wilcoxon test, depending on the equality of variances between groups, αB = 0.0125)—with sample number (angle + location) as the pairing factor—were conducted to determine whether there were statistically significant difference between PET and PLA samples. A graphical representation of the paired data is shown in Figure 10.

Analysis of the paired differences between FFF_PET and FFF_PLA samples showed that the mean values of Sa, Spk + Sk + Svk, and Ssk were statistically equal. In the case of the Sdq parameter, however, statistically significant differences were observed (*p* < 0.0001). In Figure 10, it is clearly visible that in most paired data cases, the FFF_PET sample surfaces exhibit higher Sdq values. On average, Sdq for PETG is 1.50, while for PLA it is 1.17. This is most likely due to the fact that the filament strands of PLA material are more linear, whereas the PETG strands exhibit greater waviness. This effect is particularly noticeable at higher printing angles of the sample (Figure 4c).

### 3.4. Influence of Surface Printing Angle and Measurement Location

The geometry of the model, combined with the fact that it was printed at various angles, resulted in individual surface fragments (at specific locations on the sample) being formed at different angles relative to the printer’s XY plane. This section presents the results concerning the influence of both the print angle (of a given surface relative to the XY plane) and measurement location on the surface texture parameters.

To assess the influence of factors such as print angle and measurement location on surface topography parameters (Sa, Spk + Sk + Svk, Sdq, and Ssk), an initial ANOVA analysis was performed (with sample type treated as a random effect). To satisfy the assumption of normality, the values of Sa, Spk + Sk + Svk, and Sdq were logarithmically transformed.

The results indicate that measurement angle and location (both simultaneously) did not have a significant effect on any of the examined parameters: Sa, Spk + Sk + Svk, Sdq, or Ssk. It should be noted, however, that relationships between angle and measurement location may exist for these parameters, but they should be considered separately for each sample, as described later in the article.

**Figure 11:**
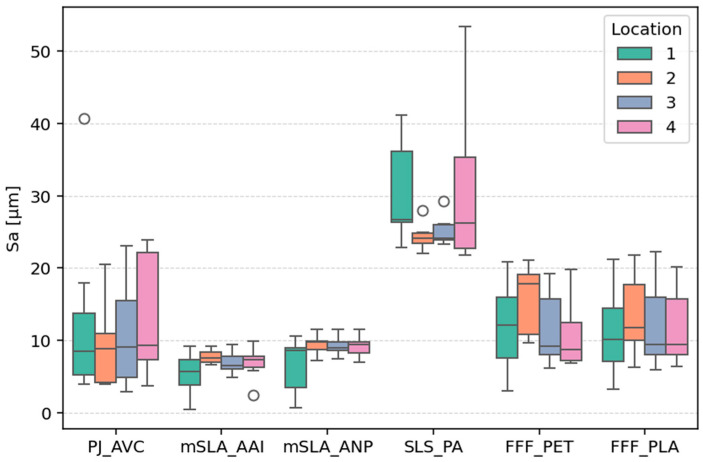
Influence of measurement location on the Sa parameter.

Next, the influence of measurement location on surface topography parameters was examined separately for each sample. ANOVA tests (with angle as a pairing factor) did not reveal a statistically significant effect of measurement location on parameter values. However, for mSLA samples, the variability of Sa and Spk + Sk + Svk at location 1, corresponding to the flat surface, was notably higher than at other locations (Figure 11).

For the mSLA_AAI sample, the standard deviation of Sa at location 1 was approximately 3.01 µm, with a coefficient of variation (CV) of 55.9%, whereas at other locations, these values ranged from 0.99 to 2.34 µm and from 12.9 to 34.2%, respectively. Similarly, for Spk + Sk + Svk at the same location, the CV was 49.2%, more than twice the variability observed at locations 2–4 (approximately 15–28%).

**Figure 12:**
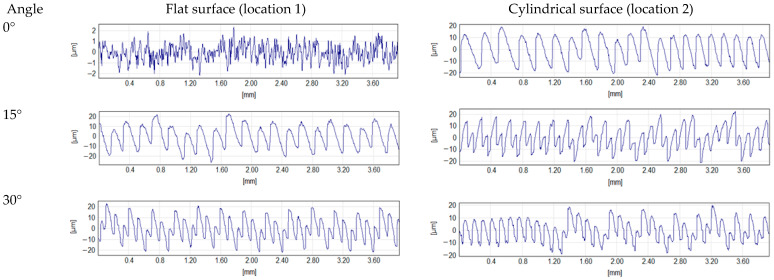
Effect of surface geometry on interlayer displacement: 2D profiles of flat and cylindrical regions in mSLA_ANP samples.

A similar trend was observed for the mSLA_ANP sample, where the CV at location 1 was 58.9% for Sa and 56.1% for Spk + Sk + Svk, while at other locations it ranged between 14–16%. This phenomenon is illustrated in Figure 12.

The greater variability observed on flat surfaces arises because, at small inclination angles relative to the printer’s XY plane, consecutive layers are only slightly shifted relative to each other. When combined with small layer thickness, this results in low height parameter values. In contrast, on curved surfaces, the relative displacement of layers is always more pronounced, significantly increasing Sa and Spk + Sk + Svk values.

Preliminary analysis showed that, in many cases, the relationship between the printing angle and the investigated parameter values could be approximated using a third-degree polynomial. To satisfy the assumptions of normally distributed residuals (assessed with the Shapiro–Wilk test, SW) and homoscedasticity (assessed with the Breusch–Pagan test, BP), all models were fitted using three forms of the dependent variable: untransformed, logarithmically transformed, and Box–Cox transformed.

After determining for which samples and parameters the third-degree model was statistically significant, the appropriateness of this degree was also evaluated. The Akaike Information Criterion (AIC) was used for this purpose. For each combination of sample type and parameter, AIC values were calculated for nested models: linear (first-degree), quadratic (second-degree), and cubic (third-degree). All models were fitted to the same data using the same variable transformations. The model with the lowest AIC, providing the best compromise between fit and complexity, was considered optimal. Table 3 summarizes the regression models that were statistically significant and had the lowest justified complexity. Table 3 summarizes the ANOVA *p*-values for each regression, testing model significance, along with the coefficient of determination (R^2^).

**Figure 13:**
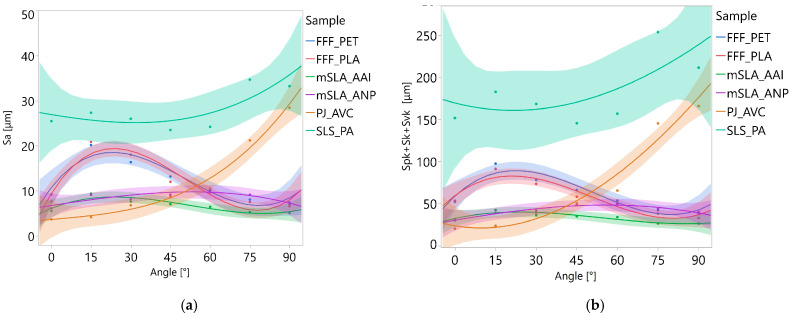
Influence of print angle on the Sa (**a**) and Spk + Sk + Svk (**b**) parameters. Points represent mean values from 4 measurement locations; lines show the cubic regression with 95% CI.

**Figure 14:**
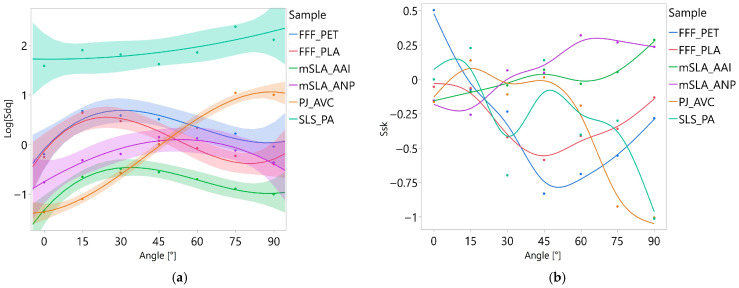
Influence of print angle on the Sdq (**a**) and Ssk (**b**) parameters. Points represent mean values from 4 measurement locations. In panel (**a**), lines show the cubic regression with 95% CI. In panel (**b**), lines are not regression curves and are included for improved readability.

The graphical presentation of the relationship between the printing angle and each surface texture parameter for the different samples is shown in Figure 13 and Figure 14. Although the optimal polynomial degree for each combination of parameter and material was determined based on the AIC, the curves in the graphs were uniformly presented as third-degree polynomials. This uniform presentation served purely illustrative purposes and ensured visual consistency across the figures, especially since the shapes of second- and third-degree curves differed only slightly within the analyzed range of angles. While regression models were fitted using either logarithmically transformed or untransformed dependent variables, the graphical presentation was always based on untransformed data to facilitate interpretation. This approach makes the results more intuitive, as the surface texture parameters have a direct geometric meaning, whereas interpreting relationships on a logarithmic scale would be less straightforward for the reader.

However, for the Sdq parameter (Figure 14), logarithmically transformed values are shown to improve readability. Regression curves were not presented for the Ssk parameter because most models were not statistically significant, and including confidence intervals would have cluttered the graphs.

For SLS samples, no statistically significant dependence of parameter values on the print angle of the surface was observed. Analysis of the topography maps also showed that the examined surfaces exhibited a similar character. The geometric structure of these samples is not dominated by the layered construction of the model—individual layers are not visible, and the fused-grain pattern prevails, resulting in an isotropic surface.

At the other end of the spectrum are PJ samples, for which the effect of print angle is strongly visible. The regression curves determined for Sa, Spk + Sk + Svk, and Sdq are well to very well fitted to the empirical data (R^2^ = 0.94, 0.94, and 0.98, respectively). This indicates that changes in print angle alone do not explain only 2–6% of the variability in the results. The regression curves for these parameters are approximately monotonic: as the print angle increases, the values of the analyzed parameters also increase. In other words, the greater the angle at which a surface was printed, the higher the roughness height and slope steepness.

The course of Ssk for PJ samples is likely strongly influenced by the formation of deep longitudinal valleys on the surfaces. At angles of 0–45°, such valleys are very few or absent, and Ssk values remain similar. From 60° onwards, the number of valleys increases, causing a sharp decrease in Ssk.

For both types of mSLA samples, the relationship between print angles and the parameters Sa and Spk + Sk + Svk was not statistically significant. For the Sdq and Ssk parameters, the developed regression equations were statistically significant; however, the fit of these curves to the empirical data can be considered only moderate. The steepness of the roughness slopes (Sdq) was lowest at surface angles of 0° and 90°, with the maximum values occurring at intermediate angles, i.e., 30–60°. The Ssk parameter exhibited a more monotonic trend, increasing as the inclination of the printed surface relative to the XY plane increased.

For FFF_PET samples, statistically significant relationship was found between print angle and the parameter Spk + Sk + Svk. For FFF_PLA samples, a significant relationship was observed for Sa. The parameters Sa and Spk + Sk + Svk are strongly correlated with each other (the Pearson correlation coefficient for these is 0.93), and tests on paired data did not show any statistically significant differences between the PLA and PET samples. Therefore, it could be assumed that the relationships for the FFF_PLA and FFF_PET samples would be similar. Their similarity is indeed visible in the plots shown in Figure 14. The lack of statistically significant models for FFF_PET and the Sa parameter, as well as for FFF_PLA and the sum of the Spk + Sk + Svk parameters, was due to the presence of individual outlying observations. However, due to the small amount of data, these were not removed from the dataset. The obtained results suggest, nevertheless, that increasing the number of measurement areas could contribute to the development of statistically significant models.

**Figure 15:**
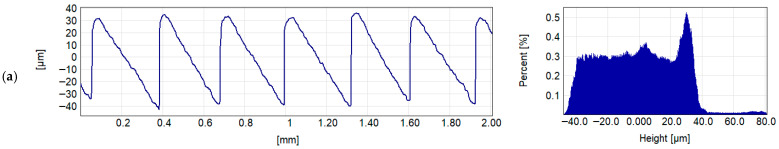

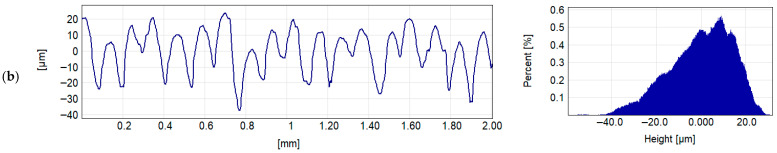
Two-dimensional surface profiles (2 mm length) and height distributions of samples printed at 15° (**a**) and 45° (**b**).

The graphs in Figure 12 and Figure 13 show a trend in which the highest roughness heights occur at angle of 15°, while the lowest values are observed at 0° and 75–90°. The greatest roughness developed when a given surface fragment contained relatively few layers. For example, reproducing the flat surface of the FFF_PLA model at a 15° angle over a 4 mm section required approximately 14 layers, whereas at a 45° angle, 36 layers were needed. With fewer layers, the spacing between irregularities is larger, and their height is greater (Figure 15) compared to surfaces formed from more layers. The variation in Sdq with print angle for both FFF samples is correlated with the trends in Sa and Spk + Sk + Svk (Figure 14a). The lack of statistically confirmed relationships in this case resulted from the absence of heteroscedasticity of the residuals. This was due to the high variability of the Sdq parameter at a 0° angle, and to a lesser extent also at 15° and 30°. In Figure 14b, a parabolic trend in the variability of the Ssk parameter depending on the angle is visible for both types of FFF samples. The lowest value of this parameter is observed at a 45° angle. To illustrate the cause, distributions of ordinates are shown in Figure 15. On the surface printed at a 45° angle, peaks dominate to the greatest extent. The reason why statistically significant models were not developed is similar to the case of Sdq: the Ssk parameter also exhibited high variability on surfaces associated with a 0° angle. In the future, increasing the sample size may help establish a significant relationship between the angle and Ssk.

## 4. Discussion

The relationship between model orientation and surface roughness is one of the key issues in AM, especially in the context of polymer models [37,38,39,40]. This influence varies depending on the technology used: MEX [37], VPP [38], MJ [39], and PBF [40]. For functional parts, the orientation often has to be selected so that the critical surfaces (e.g., mating, sealing) have the lowest possible roughness, even at the expense of extending the 3D printing time or increasing the amount of support structures. Therefore, choosing the optimal model orientation is not a simple task, as it directly affects the part’s strength, functional, and esthetic properties, as well as the cost and time of manufacturing. In the presented research, a test model was developed to assess the influence of changing the model’s orientation in the 3D printer space on surface roughness parameters.

One of the reasons for the influence of orientation on roughness in AM technologies is the staircase effect [41,42,43,44]. This effect occurs because curves and inclined surfaces are approximated by the stepwise layering of layers with a finite thickness (Δz). Its influence is particularly noticeable in FFF method. Despite the application of new solutions to minimize this effect, it still plays a crucial role in the process of assessing the surface roughness of models made using the FFF method [45,46]. Considering the research presented in the article, it was observed that the surface roughness behavior for PLA and PETG material is generally similar across the measured angular range. Since the layer thickness (Δz) was maintained at a constant 0.08 mm for both materials, the variations observed are primarily driven by the distinct rheological and thermal properties of the polymers. Typically, surfaces parallel to the build platform exhibit the lowest surface roughness values. The roughness values for both materials reached their lowest levels at the horizontal orientation (0°) and at the 75°. For PLA, the Sa value at 0° (5 µm) is little smaller to the values at 75° (8 µm). In the case of PETG, the Sa values is comparable at 0° and at 75°. These differences are attributed to the material-specific rheology, which affects the efficiency of layer flattening and fusion (bonding). Although the literature generally considers a 45° angle to be the least favorable orientation for FFF method [41], the highest surface roughness parameter values (Sa and Spk + Sk + Svk) were observed on surfaces fabricated at 15° and 30° for both PLA and PETG materials. This finding was directly influenced by the staircase effect, which is most pronounced at these specific angular settings for the parameters used. The roughness values distinctly decrease for angles above 45°, reaching local or global minima at 75°. In the case of vertical surfaces (90°), the roughness is higher than for the analyzed surface fabricated at 75°. In this instance, the value of the roughness parameters may primarily have been dominated by non-ideal layer alignment (aliasing) and non-uniformity along the *z*-axis. The high Sa parameter values for PLA and PETG material (Sa up to 20 µm) are consistent with general observations in the literature [47,48], confirming the necessity of post-processing for functional applications. A unique finding of our study, which differs from common assumptions (indicating 45° as the least favorable angle), is the observation of the maximum Sa parameter value on surfaces inclined at 15° and 30°. Although the literature focuses on 45°, it confirms that the influence of the stair-stepping effect on surface quality depends on the mutual ratio of layer thickness to path width [49]. Our results suggest that, for the constant layer thickness of 0.08 mm, the 15°/30° angles generate the greatest geometric instabilities of the layers, which should be considered when optimizing print time and orientation [50]. Additionally, the analysis of Spk reveals the existence of large, fragile peaks created during the FFF 3D printing process, resulting from inter-bead voids or poorly adhered bead edges. A high Spk value corresponds to these sharp features, predicting a severe and prolonged running-in period during which the coefficient of friction (CoF) remains unstable until these peaks are worn away. The parameter Ssk further differentiates the functional quality: PLA maintains a functionally favorable surface profile (Ssk < 0) across all angles, indicating a dominance of valleys conducive to lubricant retention. In contrast, PETG’s initial positive Ssk (15° and 30°) indicates a temporary dominance of peaks, suggesting that PETG is more susceptible to generating fragile, peaked features at minimal inclination angles, which would lead to a more severe running-in period in early use and higher initial CoF. The lowest (most negative) Ssk values near 45° for both materials suggest an optimal geometry for hydrodynamic lubrication at this angle. The wear rate and CoF in the FFF method are significantly dependent on layer thickness and infill density, which directly determine the structural quality of the part’s core. Analyzing the results more broadly, surface directionality and layer-dependent roughness are dictated by the rheology of the extruded polymer (bead shape and fusion) and the cooling rate (heat transfer). Considering the results obtained, it can be concluded that the roughness of models made using the FFF method is high and strongly dependent on the material used, even when key process settings, such as layer height (Δz), are constant. The functional differences (Ssk) and overall roughness level are primarily attributed to their distinct thermal and rheological properties. The dominance of directional patterns (layer marks) indicates that chemical or mechanical post-processing is necessary to remove this undesirable characteristic.

Considering mSLA method, the staircase effect was also observed to influence surface roughness, but due to the applied layer thickness of 0.05 mm, it is less noticeable than in FFF method [51]. For 3D printing samples using the mSLA method, two types of acrylic resin were used. The change in material type affected the variability of the roughness results. Preliminary analysis showed that samples made from the Phrozen Neon Pumpkin material had higher surface roughness parameter values than samples made from the Phrozen Aqua-Ivory 4K material. Variability was also noted in parameters describing the height and steepness of irregularities (Sa, Spk + Sk + Svk, Sdq), but no significant influence of the material change on the height distribution asymmetry (Ssk) was observed. In the conducted research, it was found that surfaces printed parallel to the platform (0°) exhibit the lowest roughness parameters. The surface roughness of the final layer was mainly dependent on the curing parameters and the accuracy of the optical system [52]. For the vertically oriented surface (90°), the parameter values are slightly higher. The increase in surface roughness parameters may result from the applied 3D printing layer thickness and errors in the layering process itself. For both material types, the highest roughness parameter values were observed for the sample angular positions in the range from 1° to 89°. This fact results from the increase in the staircase effect due to the increase in the surface’s tilt angle relative to the *z*-axis. The highest roughness values were obtained for sample angular settings oriented at 15° for the Phrozen Aqua-Ivory 4K material and 45° for the Phrozen Neon Pumpkin material. However, comparing the results obtained for samples made using mSLA method, they were characterized by the lowest variability of results compared to the other technologies selected in the research process.

The Sa values for mSLA samples (6.72–8.54 µm) and the low slope gradient (Sdq = 0.48–0.89) confirm the literature thesis [53] that mSLA offers the highest surface quality among all AM technologies for polymers. The low Spk + Sk + Svk parameter (33.36–42.16 µm) is evidence of a stable microstructure, which suggests minimal initial wear in tribological applications [54]. Additionally the extremely low Sa and the gentle surface gradient Sdq suggest that mSLA surfaces are inherently stable, require minimal or zero wear during the running-in phase, and offer excellent sealing properties. Low Spk values indicate a minimal presence of easily removable protruding peaks, which is ideal for minimizing initial wear. Despite minor variability in the obtained surface roughness parameter values compared to other additive manufacturing methods, a subtle difference is noticeable between the two resins. This difference may stem from the chemical composition of the photopolymer resins. Unfortunately, manufacturers do not disclose this data, treating it as a trade secret. Although we lack the exact composition (type and concentration of monomers, photoinitiators, pigments), the differences in roughness between the two resins can be mechanistically explained. Considering the Phrozen Neon Pumpkin resin, it possesses an intense, opaque color that contains a high concentration of pigments. These pigments strongly absorb and scatter UV light. This results in a drastic reduction in the adequate cure depth and generates sharper, more defined boundaries between the cured and uncured layers during 3D printing [55]. Consequently, intense pigmentation can contribute to the stair-stepping effect. In the case of very light, white, or ivory-colored resins (such as the Aqua-Ivory Resin), the pigments generally absorb UV light less effectively. This increases the depth of light penetration, characterized by a reduction in the stair-stepping effect, which leads to lower average roughness. This means that surfaces printed with the Aqua-Ivory resin are generally smoother and have more rounded peaks and valleys than those from resins with a very sharp curing boundary.

Considering the rheological aspect, in the case of the mSLA method, the low viscosity of the liquid resin allows for optimal self-leveling during the coating process, minimizing irregularities. The absence of a significant thermal process eliminates thermal deformation. Roughness is defined solely by the XY resolution and the internal photopolymerization effect, leading to the lowest 6.72–8.54 µm. Achieving an Sa parameter value in the range of 6.72 µm to 8.54 µm also has significant functional importance. Considering adhesion and bonding, the results obtained in the article fall within the roughness range 5–15 µm This range is often optimal for many standard adhesives as it increases the effective surface area for bonding. Consequently, mSLA components may eliminate the need for intensive surface preparation before gluing. Regarding sealing and assembly fit, the results obtained for samples fabricated using the mSLA method are much closer to the requirements for clearance fits and precise assembly, where mounting tolerances often require Sa < 5 µm. In the case of friction and wear, despite the relatively low values, mSLA surfaces still require post-processing (polishing or grinding) for critical tribological applications (e.g., bearings), where a roughness on the order of Sa < 1 µm is required. Considering the results obtained, the mSLA method provides the best surface quality immediately after 3D printing. Objects made using the mSLA method minimize the need for further finishing in applications where smoothness is critical. However, if surface treatment is necessary, it is limited to light polishing or grinding to achieve optical smoothness. The costs and processing time are the lowest among all other methods.

In the case of the SLS method, the primary influence on surface roughness is the powder granularity (incompletely melted powder particles adhering to the surface) and, to a lesser extent, the staircase effect. The roughness is more uniform, but downskin surfaces may be less well-finished due to contact with the powder and thermal effects [56]. The used SLS method allows for obtaining a more isotropic surface structure. During the research conducted, it was observed that surfaces inclined at a small angle of 15° or 30° (excluding surfaces in contact with supports or the platform, which may require post-processing) exhibit the lowest roughness. The surface is smoother, which may result from the quality of the laser weld and the powder being able to adhere better to itself in the XY plane. A significant increase in surface roughness characterizes surfaces inclined at an angle above 45°. These surfaces show a substantial increase in roughness due to the phenomenon of partially sintered powder particles “falling into” the melting laser path. As the laser moves diagonally, the heat can partially sinter the loose powder adhering to the surface, creating a grainy, irregular texture effect. For vertical surfaces (90°), it was observed that the roughness parameters are the highest. This may be primarily related to the size of the powder particles and, to a lesser extent, may result from the “staircase” effect [57]. The obtained results confirm that when analyzing samples made with SLS method, the horizontal orientation is not always the best for minimizing roughness parameters [58]. However, when interpreting the roughness parameters, it should be emphasized that when verifying surfaces made with SLS method, the most significant variability in measurement results occurred, mainly due to the granular structure of the test sample’s surface.

The obtained Sa value of 27.86 µm for PA2200 is a typical and expected value for as-built SLS components, according to reviews [59]. This value falls within the standard range of 20 µm to 35 µm and is a function of the granularity of the powder used and the sintering conditions [60]. High roughness and steep slopes Sdq indicate high local stress concentrations at the asperity tips. The high Spk parameter associated with SLS surfaces reflects a large number of loose or partially sintered particles, which are prone to easy detachment and act as abrasive wear debris during the running-in phase [60]. Optimization of energy density can improve particle fusion, which in turn reduces Spk and limits the quantity of weakly bonded particles on the surface. Analyzing the results more broadly, the high Sa value (27.86 µm) and steep slopes (Sdq = 7.67) are a direct consequence of the powder bed rheology and heat transfer during sintering. This confirms that roughness is intrinsically linked to the size of the powder particles and the unstable rheological state of the polymer during partial melting and subsequent consolidation. Comparing the obtained roughness values from the perspective of functional properties related to friction and wear, it can be concluded that models produced by the SLS method require significant post-processing to reduce roughness. Additionally, regarding esthetic aspects and optical properties, the excessive value of the roughness parameters makes the surface too coarse to meet these standards (which typically require Sa < 2 µm). This process is suitable only where simple blasting is required or when surface finish is not a priority.

Considering adhesion and assembly, an Sa parameter value of approximately 28 µm is often too high for achieving precise assembly fit and may even be too great for optimal adhesion if the adhesive is unable to effectively fill such large irregularities. Considering the results obtained, it can be concluded that models made using the SLS method is characterized by the roughest surface (as-printed), which means that in most industrial applications, intensive post-processing is necessary to reduce roughness. The high roughness values obtained necessitate aggressive material removal or the use of advanced techniques such as vibratory or chemical smoothing (if available for the material). Additionally, steep microstructure slopes (graininess) lead to rapid tool wear and necessitate longer machining cycles to remove sharp peaks. Thus, the costs of complete surface machining are very high.

In PolyJet method, orientation has a crucial and direct impact on surface roughness. The main factor affecting surface quality is the necessity of using support structures and the stair-stepping effect on slanted surfaces [61]. The analyzed article focused on conducting roughness tests on surfaces not in contact with the support material. As with mSLA method, the stair-stepping effect is visible, but not as distinctly as in FFF method due to the print layer thickness used, which was 0.032 mm. Considering the obtained results, the most optimal sample angle that minimizes the values of the surface roughness parameters is 15°. This may be due to the angular position distributing the stepping error across a larger number of shorter, less noticeable “steps.” As the sample’s angular position increases, the surface roughness parameters’ values increase exponentially. The highest values were achieved for the vertical orientation. This is because the boundary of each new layer forms microscopic steps resembling stairs. Although the layers are very thin, their height is the sole factor determining why the roughness in the direction perpendicular to the surface is the highest [62,63]. An interesting conclusion is that PolyJet method is more sensitive to changes in model orientation than mSLA method. Despite using a significantly smaller layer thickness of 0.032 mm compared to the mSLA method, which was 0.05 mm, the roughness value is lower only at an angular inclination of 15°. For other angular settings, the parameter values are lower for mSLA method for both types of materials.

This anomaly is widely discussed in the literature [64] and results from two factors: the higher viscosity of PJ resins (which limits self-leveling) and the discretization of the material during droplet jetting. These factors lead to pronounced anisotropy and higher sensitivity to orientation [65], which confirms our observation that PJ is more angle-sensitive than mSLA. Analyzing the results more broadly, intermediate roughness and anisotropy are a result of the rheology of the jetted micro-droplets. The higher viscosity compared to mSLA limits the self-leveling effect, while the discrete nature of the deposited droplets creates a distinct, anisotropic texture influenced by the print angle. Considering the results obtained, it can be concluded that models made using the PJ method have moderate roughness, but the key problem is significant anisotropy. This means that process design and part orientation in the working chamber are critical for achieving repeatable surface quality. For this reason, the finishing strategy (e.g., polishing) is heavily dependent on the direction in which successive layers of the 3D print are applied, which complicates the process and increases preparation time. Additionally the tribological reliability of PJ parts requires testing across multiple print orientations to account for structural anisotropy.

Surface topography analysis is crucial for application engineering because surface roughness is a function of rheological mechanisms, heat transfer, post-processing, and layered geometry. Specifically, the study took into account the influence of the stair-stepping effect, which is the main factor determining the roughness of inclined surfaces in layer-based technologies (FFF, mSLA, PJ). The following Table 4 synthesizes the obtained results, translating quantitative roughness data into practical design guidelines, indicating which method is optimally suited for specific functional requirements, considering the necessity and possibility of post-processing.

## 5. Conclusions

The novelty of the presented research lies in the comprehensive, comparative assessment of surface topography across multiple additive manufacturing technologies—FFF, mSLA, SLS, and PolyJet—using a standardized test model with both flat and cylindrical surfaces printed at seven different orientation angles (0–90°). Unlike many previous studies that focused on a single material or technology, this work systematically evaluates the combined effects of print angle, measurement location, and material type on key surface roughness parameters (Sa, Spk + Sk + Svk, Sdq, and Ssk), providing detailed quantitative insight into how layer thickness, staircase effect, and material-specific properties influence surface characteristics.

Additionally, this study introduces a methodological approach using replicated surfaces and high-resolution optical measurements to minimize material-dependent optical artifacts, allowing for more accurate comparison across technologies. The analysis of variability within individual samples, including the use of standardized z-scores and coefficients of variation, further provides a nuanced understanding of local topography heterogeneity—a topic rarely addressed in previous comparative AM research. The combination of regression modeling for angle-dependent roughness and statistical evaluation of material and location effects represents a holistic approach to optimizing part orientation and surface quality in polymer additive manufacturing.

The key findings of the conducted study can be summarized as follows:

1. mSLA samples exhibited the smoothest surfaces, with the lowest roughness parameters (Sa = 6.72–8.54 µm; Spk + Sk + Svk = 33.36–42.16 µm) and minimal slope steepness (Sdq = 0.48–0.89).
2. SLS surfaces had the highest roughness and slope values (Sa = 27.86 µm; Spk + Sk + Svk = 183.79 µm; Sdq = 7.67) due to deep pores and fused-grain aggregates, resulting in the most isotropic topography.
3. PJ samples displayed intermediate roughness (Sa = 11.65 µm; Spk + Sk + Svk = 72.1 µm) with pronounced directional effects influenced by print angle; deeper valleys contributed to high variability (CV ≈ 77–80%).
4. FFF surfaces showed directional patterns; topography parameters were similar for PETG and PLA in terms of Sa, Spk + Sk + Svk, and Ssk, whereas Sdq was significantly higher for PET (1.50) than for PLA (1.17). This is likely due to PLA strands being more linear and PET strands exhibiting greater waviness, particularly at higher printing angles.
5. Roughness depended on material and layer thickness, with PET exhibiting Sa = 20.38 µm and PLA Sa = 27.68 µm. Maximum roughness occurred at intermediate print angles (15–30°) when layer count was low.
6. Material type significantly influenced surface parameters in mSLA and for the Sdq parameter also in FFF samples, while print angle strongly affected PJ and FFF samples, particularly for features with fewer layers.
7. Measurement location had limited impact on surface parameters, except for flat regions in mSLA samples, where variability was notably higher.

Future studies should focus on FFF technology to further explore the effect of the printed surface angle on surface texture parameters. Further work is also needed on the surface topography of curved geometries, where the “stair-stepping” effect significantly influences measurement results. In such cases, the size of the measurement area may have a substantial impact on the calculated surface parameters. It would be valuable to explore the use of slicing data to determine the distribution of these steps and to develop methods for predicting surface topography based on this information. Additionally, future publications could analyze parameters Spk, Sk and Svk separately, which would allow for distinguishing the contribution of peaks and valleys to the overall surface structure, and thus provide insights into its tribological characteristics. Finally, future work should aim to optimize print orientation and process parameters to achieve the desired surface roughness while maintaining manufacturing efficiency.

## Figures and Tables

**Table 1:** Additive technologies used in the research process.

AM Processes	AM Technology	3D Printer	Commercial Material Name (Proper Name)	Main Parameters	Sample Name
Material Extrusion (MEX)	FFF	Prusa MK4 (Prusa Research, Prague, Czech Republic)	PLA (Polylactic acid)	Layer Height: 0.08 mm; First Layer Height: 0.20 mm; Nozzle (Extruder) Temperature: 215 °C (first layer), 210 °C (subsequent layers); Bed Temperature: 65 °C; Print Speed: Perimeters (Outer walls): ~25–40 mm/s; Infill: ~40–60 mm/s; First Layer Speed: 15–25 mm/s; Infill Density: 100%.	FFF_PLA
			PETG (Polyethylene terephthalate)	Layer Height: 0.08 mm; First Layer Height: 0.20 mm; Nozzle (Extruder) Temperature: 240 °C (first layer), 235 °C (subsequent layers) Bed Temperature: 85 °C Print Speed: Perimeters (Outer walls): ~20–35 mm/s; Infill: ~30–50 mm/s; First Layer Speed: 15–25 mm/s; Infill Density: 100%.	FFF_PET
Vat Polymerization (VPP)	mSLA	Phrozen Sonik Mini 4K (Phrozen, Taipei, Taiwan)	Phrozen Aqua-Ivory 4K (Acrylic) (Phrozen, Taipei, Taiwan)	Layer Height: 0.05 mm; Normal Exposure Time: 2.0–2.5s; Bottom Layer Count: 6 layers; Bottom Exposure Time: 25–35 s; Lifting Distance: 6 mm; Lifting Speed: 60 mm/min; Light-off Delay: 10 s.	mSLA_AAI
			Phrozen Neon Pumpkin (Acrylic) (Phrozen, Taipei, Taiwan)	Layer Height: 0.05 mm; Normal Exposure Time: 2.0–3.0s; Bottom Layer Count: 6 layers; Bottom Exposure Time: 20–25s; Lifting Distance: 6 mm; Lifting Speed: 60 mm/min; Light-off Delay: 12 s.	mSLA_ANP
Powder Bed Fusion (PBF)	SLS	EOS FORMIGA P100 (EOS GmbH, Krailling, Germany)	PA2200 (Polyamide 12) (EOS GmbH, Krailling, Germany)	Powder Layer Thickness: 0.1 mm; Chamber Temperature: around 176 °C; Laser Power: Contours: 16 W; Hatching (Infill): 21 W; Laser Scan Speed: Contours: 1500 mm/s; Hatching (Infill): 2500 mm/s.	SLS_PA
Material Jetting (MJ)	PJ	Eden 260V (Stratasys, Eden Prairie, MN, USA)	VeroClear (Acrylic) (Stratasys, Eden Prairie, MN, USA)	Layer Height: 0.032 mm; Print Mode: High Quality.	PJ_AVC

**Table 2:** Descriptive statistics of surface topography parameters for different sample types.

**Sa**						
**Sample**	**PJ_AVC**	**mSLA_AAI**	**mSLA_ANP**	**SLS_PA**	**FFF_PET**	**FFF_PLA**
Mean, µm	11.65	6.72	8.54	27.86	12.44	12.21
Std, µm	8.98	2.17	2.46	7.43	5.48	5.65
CV, %	77.09	32.34	28.85	26.66	44.06	46.25
**Spk_Sk_Svk**						
**Sample**	**PJ_AVC**	**mSLA_AAI**	**mSLA_ANP**	**SLS_PA**	**FFF_PET**	**FFF_PLA**
mean, µm	72.1	33.36	42.16	183.79	62.87	58.06
Std, µm	56.04	9.86	11.86	69.26	24.31	22.63
CV, %	77.73	29.57	28.13	37.68	38.66	38.97
**Sdq**						
**Sample**	**PJ_AVC**	**mSLA_AAI**	**mSLA_ANP**	**SLS_PA**	**FFF_PET**	**FFF_PLA**
mean	1.31	0.48	0.89	7.67	1.5	1.17
std	1.04	0.14	0.31	4.77	0.49	0.51
CV, %	79.68	29.01	34.26	62.22	32.67	43.28
**Ssk**						
**Sample**	**PJ_AVC**	**mSLA_AAI**	**mSLA_ANP**	**SLS_PA**	**FFF_PET**	**FFF_PLA**
mean	−0.31	0.0049	0.07	−0.26	−0.34	−0.3
std	0.53	0.17	0.26	0.98	0.46	0.29
CV, %	−171.54	3456	363.2	−376	−135.04	−95.78

**Table 3:** Results of the ANOVA for the regression analysis.

Sample	Parameter	Transformation	Degree	p_reg.	p_SW	p_BP	R^2^
mSLA_AAI	Sdq	no	3	<0.001	0.07	0.32	0.55
mSLA_AAI	Ssk	on	3	<0.001	0.12	0.50	0.47
mSLA_ANP	Sdq	no	2	0.005	0.19	0.22	0.35
mSLA_ANP	Ssk	no	3	<0.001	0.10	0.06	0.59
FFF_PET	Spk + Sk + Svk	no	3	<0.001	0.07	0.09	0.64
FFF_PLA	Sa	no	3	<0.001	0.74	0.52	0.82
PJ_AVC	Sa	log	2	<0.001	0.97	0.16	0.94
PJ_AVC	Spk_Sk_Svk	log	2	<0.001	0.51	0.42	0.94
PJ_AVC	Sdq	log	3	<0.001	0.97	0.06	0.98

**Table 4:** Correlation between surface roughness characteristics and recommended applications in investigated AM Technologies.

Method	Surface Characteristics	Suggested Practical Applications
FFF	Sa dependent on layer and direction, moderate Sa.	Prototyping, Enclosures, Support Elements (where tolerance is more important than surface finish).
mSLA	Lowest Sa (6.72–8.54 µm), smoothest microstructure (Sdq approx. 0.5)	Visible Surfaces, Sealing (requiring low permeability), Sliding Elements (low friction)
SLS	Highest Sa (27.86 µm), high slope (Sdq = 7.67), porosity	Structural Elements (no esthetic requirements), Bonded Surfaces (high Sa and Sdq promotes adhesion), Filters (controlled porosity)
PolyJet	Intermediate Sa, anisotropy, dimensional accuracy	Precise Coupling/Mating Parts, Functional Prototypes (accurate detail reproduction)

## Data Availability

The original contributions presented in this study are included in the article. Further inquiries can be directed to the corresponding author.
